# Immune-Modulation by Epidermal Growth Factor Receptor Inhibitors: Implication on Anti-Tumor Immunity in Lung Cancer

**DOI:** 10.1371/journal.pone.0160004

**Published:** 2016-07-28

**Authors:** Jin S. Im, Amanda C. Herrmann, Chantale Bernatchez, Cara Haymaker, Jeffrey J. Molldrem, Waun Ki Hong, Roman Perez-Soler

**Affiliations:** 1 Section of Transplantation Immunology, Department of Stem Cell Transplantation and Cellular Therapy, The University of Texas M.D. Anderson Cancer Center, Houston, Texas, United States of America; 2 Department of Melanoma Medical Oncology, The University of Texas M.D. Anderson Cancer Center, Houston, Texas, United States of America; 3 Department of Thoracic H&N Medical Oncology, The University of Texas M.D. Anderson Cancer Center, Houston, Texas, United States of America; 4 Division of Oncology, Department of Medicine, Montefiore Medical Center, Albert Einstein College of Medicine, Bronx, New York, United States of America; University of Parma, ITALY

## Abstract

Skin toxicity is the most common toxicity caused by Epidermal Growth Factor Receptor (EGFR) inhibitors, and has been associated with clinical efficacy. As EGFR inhibitors enhance the expression of antigen presenting molecules in affected skin keratinocytes, they may concurrently facilitate neo-antigen presentation in lung cancer tumor cells contributing to anti-tumor immunity. Here, we investigated the modulatory effect of the EGFR inhibitor, erlotinib on antigen presenting molecules and PD-L1, prominent immune checkpoint protein, of skin keratinocytes and lung cancer cell lines to delineate the link between EGFR signaling pathway inhibition and potential anti-tumor immunity. Erlotinib up-regulated MHC-I and MHC-II proteins on IFNγ treated keratinocytes but abrogated IFNγ-induced expression of PD-L1, suggesting the potential role of infiltrating autoreactive T cells in the damage of keratinocytes in affected skin. Interestingly, the surface expression of MHC-I, MHC-II, and PD-L1 was up-regulated in response to IFNγ more often in lung cancer cell lines sensitive to erlotinib, but only expression of PD-L1 was inhibited by erlotinib. Further, erlotinib significantly increased T cell mediated cytotoxicity on lung cancer cells. Lastly, the analysis of gene expression dataset of 186 lung cancer cell lines from Cancer Cell Line Encyclopedia demonstrated that overexpression of PD-L1 was associated with sensitivity to erlotinib and higher expression of genes related to antigen presenting pathways and IFNγ signaling pathway. Our findings suggest that the EGFR inhibitors can facilitate anti-tumor adaptive immune responses by breaking tolerance especially in EGFR driven lung cancer that are associated with overexpression of PD-L1 and genes related to antigen presentation and inflammation.

## Introduction

Lung cancer remains a leading cause of cancer death in the Unites States, with 158,040 estimated death to occur in 2015 [1]. Despite recent advances in multi-modality treatment strategy, the relapse rate for early stage lung cancer is significant. Only 16.8% of patients with lung cancer of all stages survive more than 5 year, and 5 year survival rate for advanced stage or metastatic lung cancer patients are dismal [2].

Epidermal Growth Factor Receptor (EGFR) Tyrosine Kinase Inhibitors (TKIs) are frontline therapy for advanced or metastatic non-small cell lung cancer (NSCLC) with sensitizing EGFR mutations such as exon 19 deletion or exon 21 L858R mutation [3]. About 10% of Caucasian and up to 50% of Asian patients with NSCLC harbor sensitizing mutations and respond to EGFR inhibitors resulting in a dramatic disease control with the improvement of symptoms. Median duration of the response ranges from 9–14 months and most patients eventually develop the resistance to EGFR inhibitors through various resistant mechanisms [4]. One of resistant mechanisms is the acquisition of the resistant mutation, T790M, and it has been reported to occur in 50% of patients after the disease progression on EGFR inhibitors [5,6].

Skin toxicity is the major toxicity associated with EGFR inhibitors including TKIs and blocking antibodies such as cetuximab or panituzumab [7–9]. Acneiform skin rash occurs up to 70–80% of patients during the course of therapy with EGFR inhibitors, and can be treated with topical steroid and antibiotics [9]. However, it often becomes severe enough to compromise the quality of life, thus results in interruption or cessation of the treatment. Interestingly, the severity of skin rash due to EGFR inhibitors has been associated with the better response rate, progression free survival, and overall survival from two large phase III clinical trials [10]. Subsequently, it has been used as a biomarker to optimize dosing of EGFR inhibitors to treat advanced NSCLC patients in recent phase II clinical trial [11].

EGFR signaling pathway is thought to play an essential role in skin repair and inflammation [12]. The blockade of EGFR signaling pathway enhances the inflammation in skin through up-regulation of chemokines, and recruits mononuclear cells including T cells, Natural Killer cells (NK), macrophages, and TRAIL-positive dendritic cells [13–17]. In addition, EGFR inhibitors have been shown to up-regulate MHC-I, and MHC-II, CIITA complex on IFNγ treated skin keratinocytes, implying the potential role of infiltrating autoreactive T cells in the damage of skin [18]. Similar immune-modulatory process by EGFR inhibitors may take place in certain malignancies. For example, EGFR inhibitors can up-regulate the expression of MHC-II and CIITA compartment on head and neck squamous cell carcinoma cell line and augment antigen specific anti-tumor T cell responses [19]. Most recently, EGFR inhibitors have been shown to down-modulate baseline PD-L1 expression, a prominent immune-checkpoint protein, on selected non-small cell lung cancer cell lines with sensitive EGFR mutations that expressed high baseline level of PD-L1 proteins [20–22]. As the PD-L1 proteins were reportedly overexpressed on selected lung cancer biopsy or surgical specimen from harboring sensitive EGFR mutations [23–25], it is possible that EGFR inhibitors can promote anti-tumor T cell responses in lung cancer via up-regulation of antigen presenting pathway while down-modulating PD-L1 expression in concurrent fashion as with skin keratinocytes.

The inhibition of PD-1 and PD-L1 axis has emerged as new immunotherapeutic with dramatic and durable responses in certain solid tumors including melanoma, non-small cell lung cancer, renal cell carcinoma etc, especially in patients with the overexpression of intratumoral PD-L1 proteins [26–29]. Co-targeting other immune check point proteins such as CTLA-4, LAG-3, and Tim-3, with blockade of PD-1/PD-L1 is a potential approach to synergize clinical responses in immunotherapy, as demonstrated by phase I clinical trial [30] and multiple preclinical studies [31–33]. Combination immunotherapy with cytotoxic chemotherapy or radiotherapy is another approach to augment anti-tumor immunity likely through several immune-potentiating mechanisms, and is being evaluated in the setting of clinical trials [34]. Lastly, several clinical trials evaluating targeted therapy such as ALK or EGFR inhibitors in non-small cell lung cancer or BRAF inhibitors in melanoma for combination immunotherapy are currently on-going, but further investigations are needed to elucidate the precise immune-mediated mechanism of action to enhance anti-tumor immunity.

Here, we investigated the effect of EGFR signaling inhibition on skin keratinocytes and various lung cancer cell lines for the expression of MHC-I, MHC-II, and PD-L1, and impact on T cell mediated tumor killing in order to delineate the link between EGFR signaling pathways to anti-tumor immunity. Lastly, we investigated the correlation of PD-L1 expression to sensitivity to EGFR inhibitors and expression of various genes critical for antigen presenting pathways using drug sensitivity and gene expression dataset of 186 lung cancer cell lines from Cancer Cell Line Encyclopedia.

## Materials and Methods

### Cells and cell culture

Human immortalized keratinocytes, HaCaT cells were grown in RPMI 1640 media (Life Technologies, Grand Island, NY) supplemented with 10% Fetal Bovine Serum (FBS, Life Technologies, Grand Island, NY). All lung cancer cell lines (Table 1) were obtained from American Type Culture Collection (ATCC, Manassas, VA), and grown in RPMI 1640 supplemented with 10% FBS.

**Table 1:** Lung Cancer Cell lines used in the evaluation of PD-L1 protein expression.

Cell Line	Histology	EGFR Mutations	EC_50_*	IC_50_*
**HCC827**	Adenocarcinoma	delE746-A750	0.02933	0.03891
**NCI-H3255**	Adenocarcinoma	L858R	0.07416	0.07517
**HCC4006**	Adenocarcinoma	delL747-A750	0.11025	0.12425
**HCC2935**	Adenocarcinoma	delE746-S752	0.18314	0.28077
**NCI-H1650**	Adenocarcinoma	delE746-A750	0.06451	8
**A549**	Adenocarcinoma	Wildtype	2.39471	8
**NCI-H441**	Adenocarcinoma	Wildtype	3.36504	8
**NCI-H2023**	Adenocarcinoma	Wildtype	8.32822	8
**NCI-H1299**	Large cell neuroendocrine	Wildtype	8.62741	8
**NCI-H1975**	Adenocarcinoma	T790M, L858R	8.81917	8
**NCI-H661**	Large cell carcinoma	Wildtype	NA	8

*EC_50_ (half maximum effective concentration) and IC_50_ (half maximum inhibitory concentration) of Erlotinib for various lung cancer cell lines have been previously reported in CCLE.

### Antibodies and cytokines

Fluorescein-conjugated Anti-HLA-A,B,C, Allophycocyanin-conjugated anti-HLA-DR, Phycoerythrin-conjugated anti-PD-L1 antibodies were obtained from BD Bioscience (San Jose, CA). Human recombinant Interferon (IFN)γ was purchased from Peprotech (Rocky Hill, NJ). Epidermal growth factor (EGF) was purchased from Life Technologies (Grand island, NY). Erlotinib was purchased from Sellect Chemicals (Houston, TX).

### Flowcytometry

HaCaT or various lung cancer cell lines were seeded in triplicates in 96 well plate a day prior to assay, and treated with IFNγ in different concentrations ranging from 1 pg/ml to 1000 pg/ml in the presence or absence of erlotinib at a concentration of 10 μM for 24 hours. Next, cells were trypsinized to a single cell suspension and stained with fluorochrome-conjugated antibodies against MHC-1, MHC-II, and PD-L1 in phosphate Buffered Saline (PBS) containing 2% FBS and 0.05% sodium azide for 30 minutes. After washing, cells were subjected to high-throughput flowcytometry in 96 well format using FACSCanto II (BD bioscience, San Jose, CA). Acquisition data were analyzed using FlowJo Software version 10 (Tree Star, Ashland, Or), and presented as mean fluorescence intensities (MFIs) ± standard deviation (SD) of triplicates for each staining for MHC-I, MHC-II, and PD-L1, respectively.

### Reverse Transcription quantitative Polymerized Chain Reaction (RT qPCR)

Half to one million of lung cancer cells or HaCaT cells were seeded in 6 well plate a day prior to experiment, then treated with IFNγ at a concentration of 100 pg/ml in the presence or absence of erlotinib at a concentration of 10 μM for 24 hours. Trypsinized cells were subjected to the isolation of a total RNA (tRNA) using RNeasy Plus Micro kit from Qiagen (Valencia, CA) according to manufacturer’s instruction. The tRNA was quantified using Cytation Cell Imaging Multi-Mode Readers (Biotek, Winooski, VT). All tRNA samples were measured as 2.0 of Optical Density (O.D.) 260:280 ratio. RNA integrity was assessed for selected tRNA, and RNA Integrity Number (RIN) ranged from 9.5 to 10. One μg of tRNA was used to generate complementary DNA (cDNA) using iScript cDNA Synthesis Kit (Bio-Rad, Hercules, CA). Next, qPCR was performed in quadruplicates to evaluate the relative expression of PD-L1 to house keeping gene, actin transcripts using SsoAdvanced Universal SYBR Green Supermix (Bio-Rad, Hercules, CA) and CFX96 Touch^TM^ Real-Time PCR Detection system (Bio-Rad, Hercules, CA). Primers used in qPCR were synthesized from Sigma-Adrich (St. Louis, MO), and include PD-L1 (forward primer 5′-TATGGTGGTGCCGACTACAA -3′, reverse primer 5′-TGGCTCCCAGAATTACCAAG-3), actin as endogenous control (forward primer 5′-TCCTGTGGCATCCACGAAAC-3′, reverse primer 5′-GAAGCATTTGCGGACGAT-3′). Data was analyzed using CFX manager^TM^ Software (Bio-Rad, Hercules, CA).

### Cytotoxic T cell assay

H441, HLA-A2^+^ lung cancer cells were seeded to 6 well plates and pretreated with or without 10 μM erlotinib in the presence or absence of MART-1 27M (AAGIGILTV) peptide or CMV pp65 (NLVPMVATV) control peptide at a concentration of 10 μg/ml for 24 hours. Next, typsinized lung cancer cells were then labeled with calcein-acetoxylmethyl (AM) (Molecular Probes, OR) at concentration of 10 μg/ml for 30 minutes at 37°C, and used as target cells after washing off unlabeled calcein-AM. Subsequently, MART-1/HLA-A2 specific cytotoxic T cells (1007G) were added at different effector;target ratios to 5000 target cells in 100 μl of complete media in quadruplicates in V-bottom 96-well plate. After incubation for 4 hours at 37°C, 50 μl of supernatant was transferred to 96-well special optics low fluorescence assay plate to measure calcein-AM release using Cytation (BioTek, VT) (excitation at 485 nm, emission at 528 nm). Spontaneous release represents calcein-AM release of target cells in media alone, and maximum release represents calceim-AM release of target cells in media containing 2% Triton X-100. Specific lysis was calculated according to following formula: [(test release-spontaneous release)/(maximum release-spontaneous release)]x100.

### Comprehensive cell line encyclopedia (CCLE) data analysis

The gene expression dataset of 186 lung cancer cell lines and drug sensitivity data of 91 lung cancer cell lines were obtained at the following site [35]: http://www.broadinstitute.org/ccle/home. Lung cancer cell lines were divided into two groups, PD-L1^high^ and PD-L1^low^ lung cancer cell lines, according to median log_2_ Robust Multi-array Average (RMA) values. Log_2_ RMA values were used to evaluate selected gene expression between PD-L1^high^ and PD-L1^low^ lung cancer cell lines.

### Statistical Analysis

All statistical analysis was performed using Prism version 6.0 (GraphPad, La Jolla, CA). Wilcoxon-Mann-Whitney test was used to compare Log_2_ RMA values of selected gene expression between PD-L1^high^ and PD-L1^low^ lung cancer cell lines, T cell mediated cytotoxicity on lung cancer cell lines treated with or without erlotinib, and surface expression of MHC-I, MHC-II, and PD-L1 of various cell lines treated with IFNγ and/or erlotinib Non-parametric Wilcoxon test was used for paired analysis to evaluate the impact of EGFR inhibitors on surface expression of MHC-I, MHC-II, and PD-L1 proteins between IFNγ or Erlotinib treated or untreated lung cancer cell lines sensitive or resistant to erlotinib.

## Results

### Erlotinib up-regulates the expression of antigen presenting molecules but abrogates PD-L1 expression on IFNγ treated human keratinocytes

In order to elucidate the immune-mechanism of skin rash caused by EGFR inhibitors, we first investigated the modulatory effects of erlotinib, one of EGFR inhibitors, on the expression pattern of antigen presenting molecules and PD-L1 on skin keratinocytes. Previously, one of malignant skin keratinocyte cell line, A431, was used to demonstrate the synergistic effects of EGFR inhibitors on the expression of MHC-I and MHC-II because this particular cell line expresses abnormally high level of EGFR compared to normal skin keratinocytes, thus the effect of EGFR inhibition would be accentuated [18,36,37]. Here, we decided to use another skin keratinocyte cell line, HaCaT cells for our analysis because they are known to express a comparable level of EGFR to primary skin keratinocyte so that we could extrapolate the modulatory effect of EGFR inhibitors observed in HaCaT cells to what might happen in primary skin keratinocytes in physiologic condition [37].

**Fig 1:**
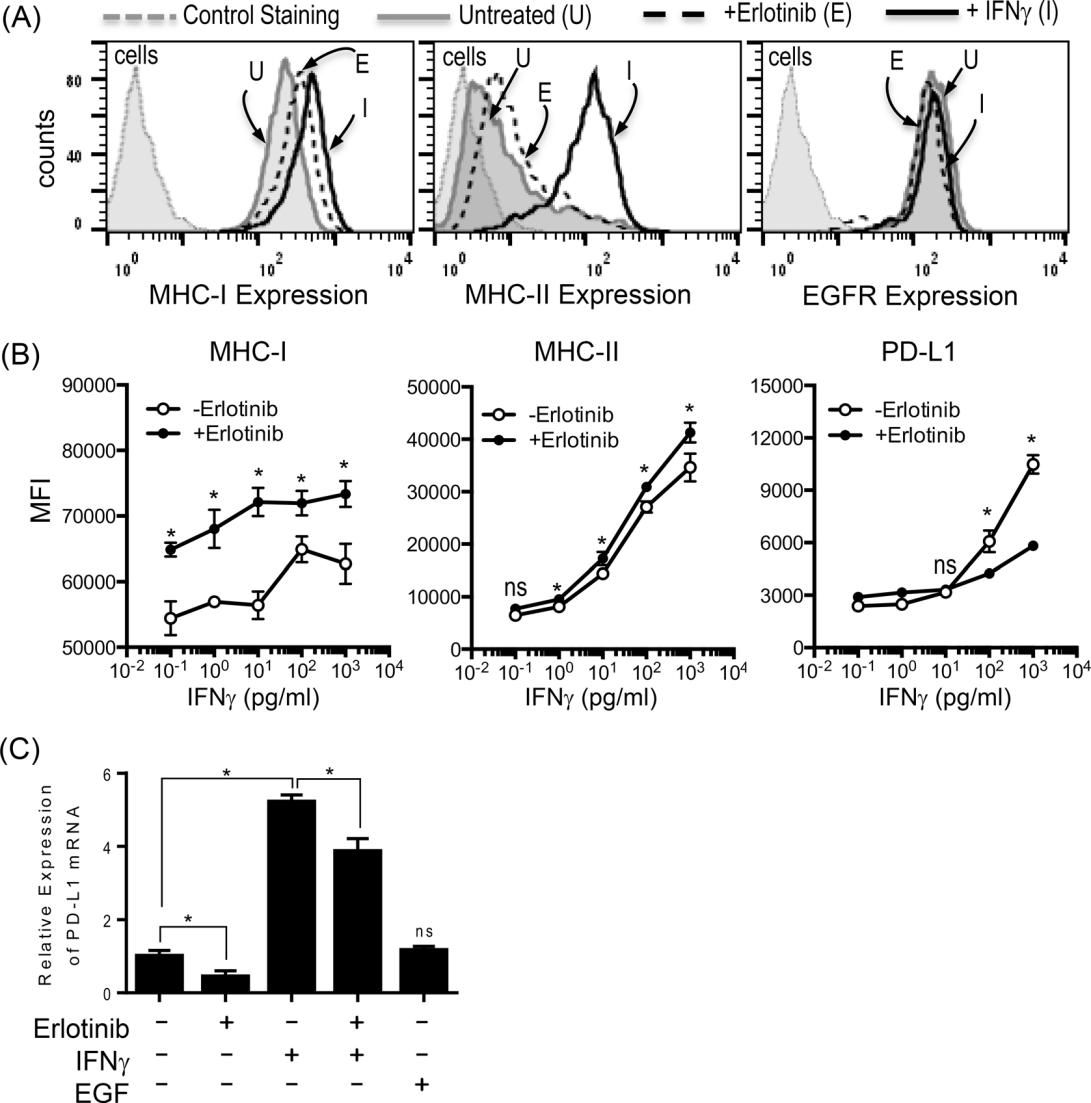
EGFR inhibitor differentially modulates the expression of antigen presenting molecules and PD-L1 on HaCaT cells. (A) HaCaT cells were treated with IFNγ at concentration of 1000 pg/ml or erlotinib at concentration of 20 μM for 24 hours, and assessed for surface expression of MHC-I, MHC-II, and EGFR. Erlotinib up-regulated the expression of MHC-I on HaCaT cells while IFNγ upregulated both MHC-I and MHC-II. Neither treatments affected EGFR expression. Gray dotted line: unstained HaCaT cells, gray solid line: untreated HaCaT cells stained for MHC-I, MHC-II, or EGFR. Black dotted line: erlotinib treated HaCaT cells stained for MHC-I, MHC-II, or EGFR. Black solid line: IFNγ treated HaCaT cells stained for MHC-I, MHC-II, or EGFR. (B) HaCaT cells were treated with IFNγ at different concentrations ranging 1 pg/ml to 1000 pg/ml in the presence or absence of erlotinib at concentration of 10 μM for 24 hours, and assessed for surface expression of MHC-I, MHC-II, and PD-L1 in Mean Fluorescence Intensity (MFI). While the expression of MHC-I and MHC-II on HaCaT cells was further up-regulated in the presence of erlotinib and IFNγ at all dose range tested for MHC-I and at a greater than 1pg/ml for MHC-II compared to IFNγ alone, IFNγ induced overexpression of PD-L1 was abrogated by the addition of erlotinib to IFNγ at a greater than 100 pg/ml. The experiment shown here was performed in triplicates, and a representative of three independent experiments.(C) A total RNA from HaCaT cells treated with or without erlotinib, IFNγ, or EGF was assessed for the relative expression of PD-L1 transcripts via Reverse Transcription quantitative Polymerase Chain Reaction (RT-qPCR) in quadruplicates. Erlotinib treatment resulted in statistically significant decrease in PD-L1 transcripts on HaCaT cells P-value less than 0.05 (*) was determined as being statistically significance. “NS” represent “statistically not-significant”.

As expected, HaCaT cells up-regulated the surface expression of both MHC-I and MHC-II with IFNγ treatment. However, only the expression of MHC-I on HaCaT cells was slightly increased by the treatment with EGFR inhibitor, Erlotinib. Both treatments did not alter the surface expression of EGFR on HaCaT cells (Fig 1A). Next, we examined the synergistic effect of erlotinib in combination with IFNγ on the expression of MHC-I, MHC-II, and PD-L1 (Fig 1B). First, all of the MHC-I, MHC-II, and PL-D1 expression were up-regulated by IFNγ treatment in a dose dependent manner. The surface expression of MHC-I and MHC-II was further augmented in the presence of erlotinib and IFNγ which confirmed the previous observations [18]. On the contrary, erlotinib abrogated IFNγ induced PD-L1 expression on HaCaT cells (Fig 1B). The down-modulation of PD-L1 by Erlotinib was at the level of mRNA transcript demonstrated by quantitative RT PCR (Fig 1C). Taken together, EGFR inhibitors can up-regulate antigen presenting proteins while down-regulating PD-L1, negative immune-regulator on skin keratinocytes in the area of inflammation, thus can promote immune-mediated damages by infiltrating autoreactive T cells in affected skin.

### Erlotinib abrogates IFNγ induced PD-L1 expression in lung cancer cell lines without adversely affecting the expression of antigen presenting proteins

**Fig 2:**
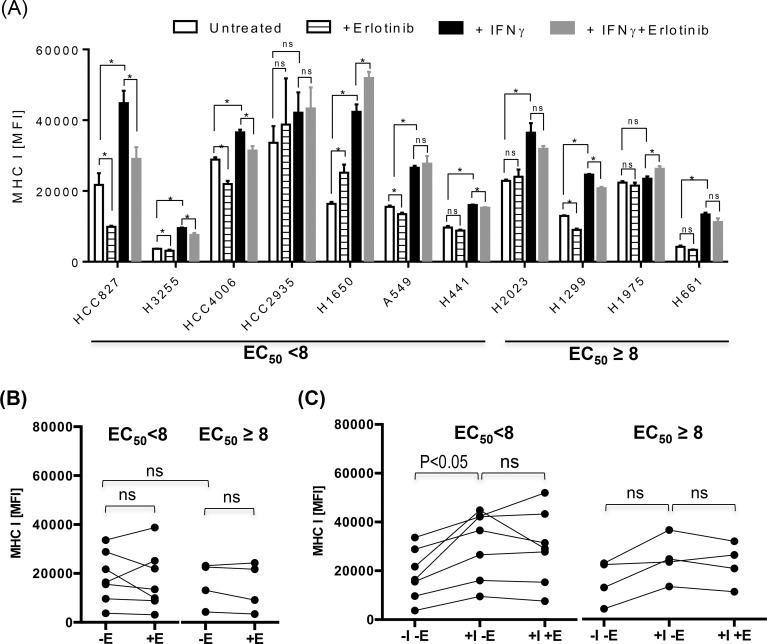
Differential effects on MHC-I in lung cancer cell lines by IFNγ and EGFR inhibitors. Various lung cancer cell lines were treated with IFNγ at a concentration of 1000 pg/ml in the presence or absence of erlotinib at concentration of 10 μM for 24 hours, and assessed for surface expression of MHC-I. (A) The average mean fluorescence intensities (MFIs) ± standard deviation (SD) of MHC-I staining were represented as bar graphs.(B and C) For further statistical analysis, lung cancer cell lines were divided into two groups according to the half maximum effective concentration, EC_50_ (EC_50_ <8 vs EC_50_ ≥ 8) of erlotinib, and average MFI of MHC I for individual cell line was represented as a single dot for further statistical analysis to assess the impact of treatment with IFNγ and/or erlotinib on MHC I expression within group of lung cancer cell lines sensitive to erlotinib (EC_50_ <8) or resistant to erlotinib (EC_50_ ≥8) There was a trend toward higher expression of MHC-I in untreated lung cancer cell lines sensitive to erlotinib (BB), and the treatment with IFNγ further up-regulated the expression of MHC-I more often on lung cancer cell lines sensitive to erlotinib compared to lung cancer cell line resistant to erlotinib (C). The addition of erlotinib to untreated or IFNγ treated lung cancer cell lines did not significantly altered the expression of MHC-I in most cell lines regardless of sensitivity to erlotinib (B and C). Abbreviations used in this experiment are as follow, E: erlotinib, I: IFNγ. The experiment shown here was performed in triplicates, and a representative of three independent experiments. P-value less than 0.05 was determined as statistically significance. “NS” represent “statistically non-significant”.

**Fig 3:**
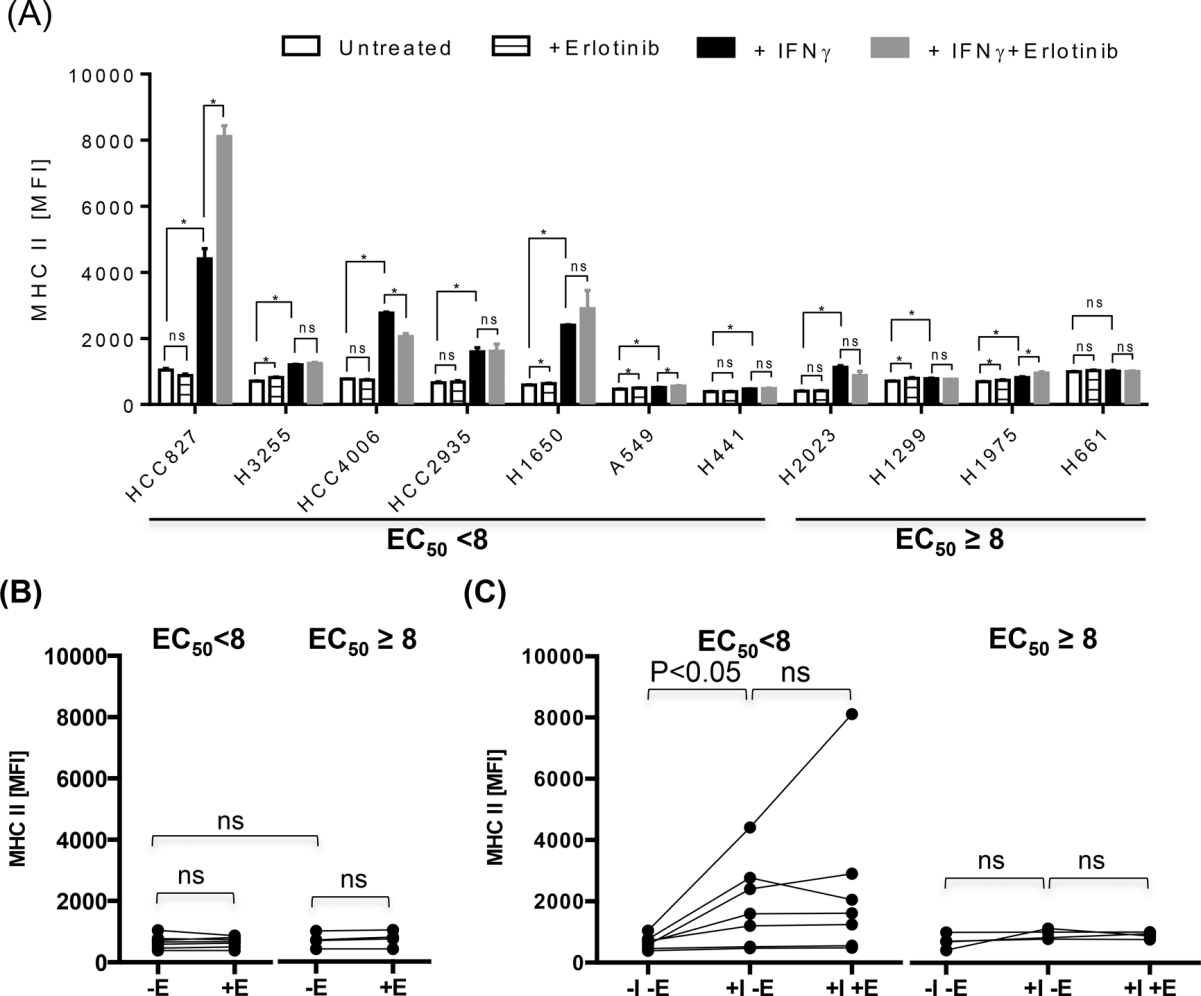
Differential effects on MHC-II in lung cancer cell lines by IFNγ and EGFR inhibitors. Various lung cancer cell lines were treated with IFNγ at a concentration of 1000 pg/ml in the presence or absence of erlotinib at concentration of 10 μM for 24 hours, and assessed for surface expression of MHC-II. (A) The average MFI ± SD of MHC-II staining were represented as bar graphs. (B and C) Lung cancer cell lines were divided into two groups according to the half maximum effective concentration, EC_50_ (EC_50_ <8 vs EC_50_ ≥ 8) of erlotinib, and average MFI of MHC-II for individual cell line was represented as a single dot for further statistical analysis to assess the effect of IFNγ and/or erlotinib on the expression of MHC-II. There was only minimal baseline expression of MHC-II in untreated lung cancer cell lines regardless of sensitivity to erlotinib (B), but the treatment with IFNγ significantly up-regulated the expression of MHC-II more often on lung cancer cell lines sensitive to erlotinib compared to lung cancer cell line resistant to erlotinib (C). The addition of erlotinib to untreated or IFNγ treated lung cancer cell lines did not significantly altered the expression of MHC-II in most cell lines (B and C). Abbreviations used in this experiment are as follow, E: erlotinib, I: IFNγ. The experiment shown here was performed in triplicates, and a representative of three independent experiments. P-value less than 0.05 (*) was determined as being significant, “ns” represents “not statistically significant”.

**Fig 4:**
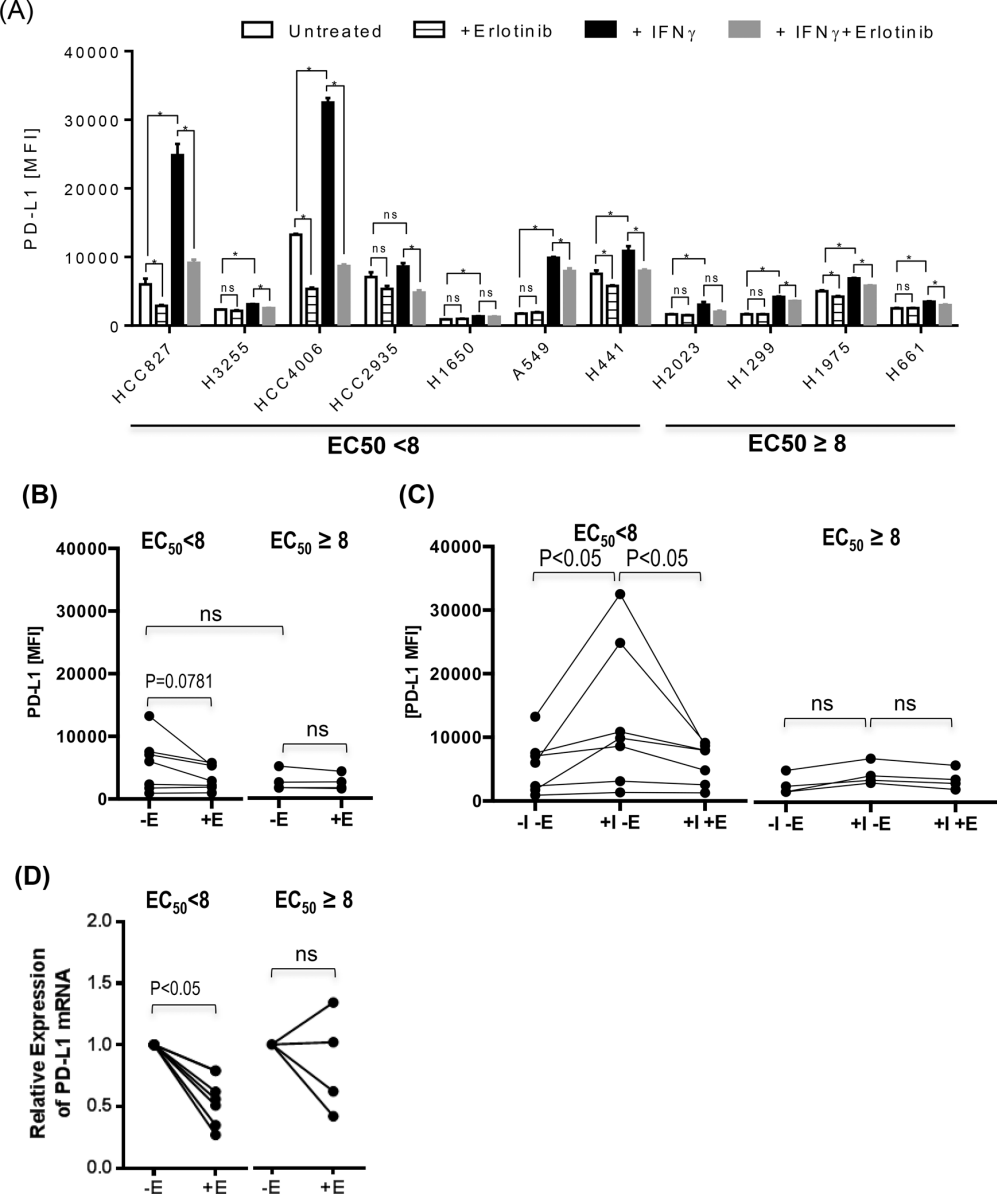
EGFR inhibitor down-regulates the PD-L1 expression on lung cancer cell lines. Various lung cancer cell lines were treated with IFNγ at a concentration of 1000 pg/ml in the presence or absence of erlotinib at concentration of 10 μM for 24 hours, and assessed for surface expression of PD-L1. (A) The average MFIs± SD of PD-L1 staining were represented as bar graphs in (A) (B and C) Lung cancer cell lines were divided into two groups according to EC_50_ (EC_50_ <8 vs EC_50_ ≥ 8) of erlotinib, and average MFI of PD-L1staining for individual cell line was represented as a single dot in dot plots for further statistical analysis to assess the influence of IFNγ and/or erlotinib on PD-L1 expression. There was a trend toward higher expression of PD-L1 in untreated lung cancer cell lines sensitive to erlotinib, and erlotinib treatment down-regulated PD-L1 expression on lung cancer cell lines sensitive to erlotinib but not statistically significant (B). The treatment with IFNγ significantly up-regulated the expression of PD-L1 proteins on lung cancer cell lines sensitive to erlotinib, and the addition of erlotinib significantly inhibited IFNγ-induced PD-L1 overexpression on lung cancer cell lines sensitive to erlotinib (C). Abbreviation used in this experiment is as follows. E: Erlotinib, I: IFNγ. The experiment shown here was performed in triplicates, and a representative of three independent experiments. (D) Various lung cancer cells were cultured in the presence or absence of erlotinib at a concentration of 10 μM for 24 hours, and the total RNA was isolated for Reverse Transcription quantitative Polymerase Chain Reaction (RT-qPCR) performed in quadruplicates to evaluate the expression of PD-L1 transcripts. The relative PD-L1 expression of individual lung cancer cell line treated with erlotinib to untreated control was represented as a single dot. Lung cancer cell lines were again divided into two groups according to EC_50_ (EC_50_ <8 vs EC_50_ ≥ 8) of erlotinib. Erlotinib down-regulated PD-L1 expression at the transcript level, and inhibitory effect was more pronounced in lung cancer cells sensitive to EGFR inhibitors. P-value less than 0.05 was determined as statistically significance. “NS” represent “statistically non-significant”.

Pollack at al has previously reported that EGFR inhibitors synergistically up-regulated the expression of antigen presenting proteins in one of squamous cell carcinoma (SCC) of head and neck cancer cell lines among other cell lines originated from various cancer such as melanoma or colon cancer [18]. To our knowledge, the potential modulation of antigen presenting molecules by EGFR inhibitors has not been demonstrated in the setting of lung cancer despite EGFR inhibitors are most commonly used in the treatment of lung cancer among other malignancies. Therefore, we assessed a series of lung cancer cell lines with wide range of known sensitivity to erlotinib (Table 1) for the impact of EGFR inhibitors on MHC-I, MHC-II, and PD-L1 expression in the presence or absence of inflammatory cytokine, IFNγ (Figs 2, 3 and 4).

First, there was a trend towards a higher expression of PD-L1 and MHC-I in lung cancer cell lines sensitive to erlotinib (EC_50_ <8) compared to those resistant to erlotinib (EC_50_ ≥8), but it was not statistically significant likely due to the heterogeneity of lung cancer cell lines and small number of lung cancer cell lines tested (Figs 2B and 4B). There was a minimal expression of MHC-II in all lung cancer cell lines tested, and this is anticipated as MHC II genes are not typically expressed in non-hematopoietic cells without an inflammatory signal such as IFNγ (Fig 3B). The treatment with IFNγ up-regulated the surface expression of MHC-I, MHC-II, and PD-L1 more frequently on lung cancer cell lines sensitive to erlotinib than on those resistant erlotinib (Figs 2C, 3C and 4C), suggesting that EGFR-driven lung cancer may be immunologically reactive.

Erlotinib altered the expression of antigen presenting molecules on several lung cancer cell lines tested but there was no consensus trend towards either up-regulation or down-regulation likely again due to the heterogeneity of lung cancer tumor cell lines (Figs 2C & 3C). For example, HCC2935, H1650, and H1975 up-regulated the expression of MHC-I upon the treatment with erlotinib in the presence of IFNγ, while HCC827 and H1650 potentiated the MHC-II expression. In contrast, erlotinib uniformly down-regulated baseline PD-L1 expression on untreated lung cancer cell lines sensitive to erlotinib, although it was not statistically significant (Fig 4B, p = 0.0781). In addition, it significantly inhibited the IFNγ induced overexpression of PD-L1 proteins especially in EGFR driven lung cancer cell lines (p<0.05) (Fig 4C). Further analysis using quantitative RT-PCR confirmed that the down-regulation of PD-L1 expression by erlotinib was at the level of gene transcription (Fig 4D).

### EGFR inhibitors enhance T cell mediated tumor killing

Although EGFR inhibitor, an effective therapy for EGFR driven lung cancer, has been shown to down-modulate PD-L1 on lung cancer cell lines in our study and other reports [20,21], whether it leads to better T cell mediated tumor killing has not been clearly demonstrated. Previously, Chen at al attempted to show improved tumor killing using co-culture system with HCC827 as target tumor cells and peripheral blood mononucleate cells (PMBC) from random donor and did not observed improved tumor killing by alloreactive T cells when a combination therapy with PD-1/PD-L1 blockade and EGFR inhibitors was used [20]. This was likely due to the difficulty to modulate the cytotoxic function of less well-defined alloreactive T cells. Thus further investigation is needed to evaluate EGFR inhibitors for modulating T cell mediated tumor killing in antigen specific manner.

**Fig 5:**
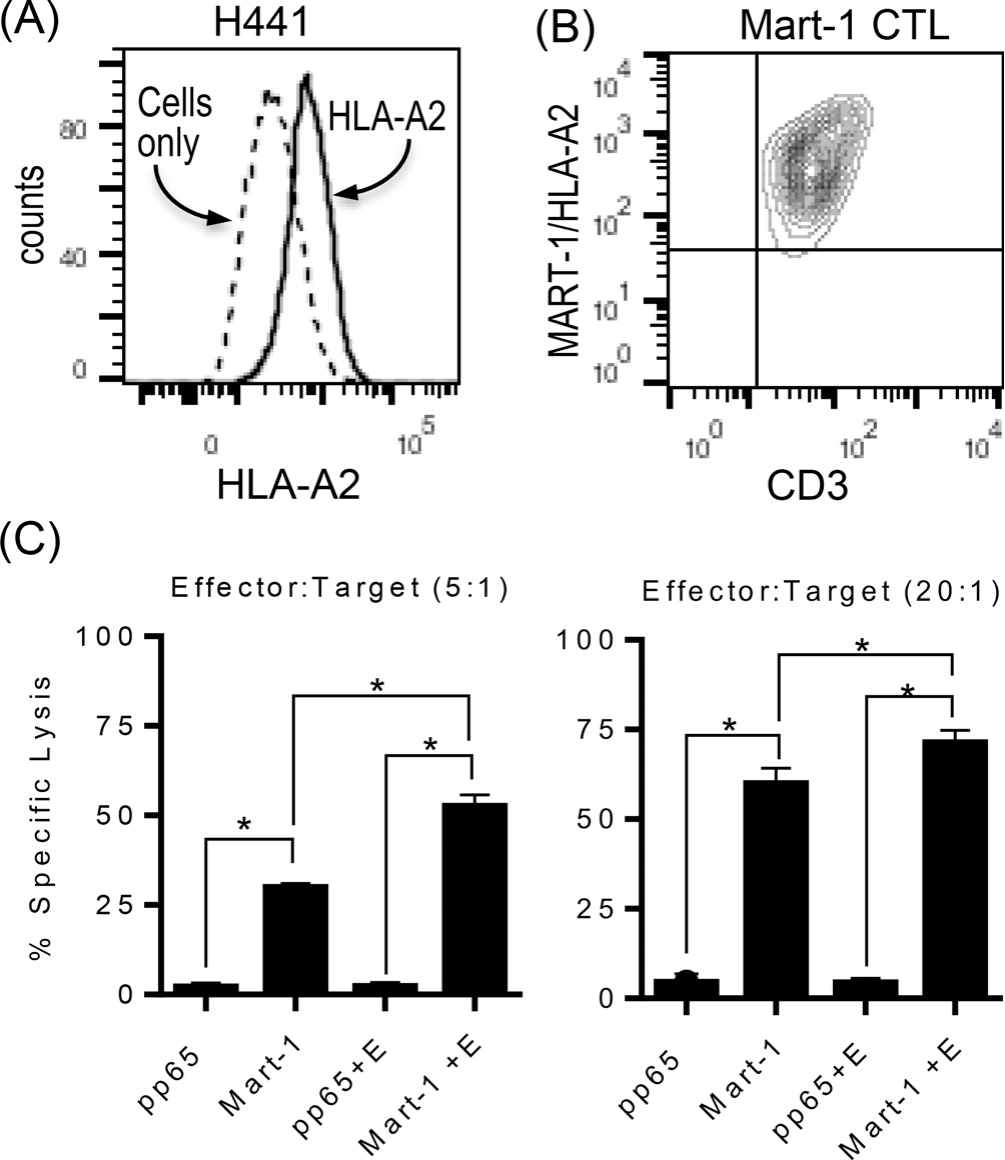
EGFR inhibitor increases antigen specific T cell mediated tumor killing. (A) The expression of HLA-A2 on HLA-A2^+^ lung cancer cell line, H441, was confirmed using flowcytometry. (B) Mart-1 (M27) specific cytotoxic T cell line (CTL), 1007G, was stained with anti-CD3 and Mart-1 (M27)/HLA-A2 tetramer to confirm the antigenic specificity. More than 90% of T cells expressed T cell receptors specific for Mart-1(M27)/HLA-A2 tetramers. (C) H441 cells pretreated with or without erlotinib in the presence or absence of Mart-1(M27) or pp65 control peptide were used as target cells for standard cytotoxic T cell (CTL) assay using Mart-1(M27) specific CTLs as effector T cells. Percent Specific cytotoxicity was calculated as follows: [(test release-spontaneous release)/(maximum release-spontaneous release)] × 100. Mart-1 (M27) specific CTL efficiently lysed H441 only in the presence of Mart-1 peptide but not control peptide, and the treatment of erlotinib significantly increased T cell mediated killing. The experiment shown here was performed in quadruplicates, and a representative of two independent experiments.

Therefore, we decided to take advantage of already established cytotoxic T cell line (CTL) specific for MART-1 (M27) peptide restricted to HLA-A2 as effector T cells (Fig 5B). HLA-A2^+^ lung cancer cell line, H441, was chosen as target lung cancer cells as H441 expressed a detectable level of PD-L1 proteins that were down-modulated by EGFR inhibitor (Fig 2A and Fig 5A). H441 lung cancer cells were pretreated with or without EGFR inhibitor in the presence of MART-1 (M27) peptide or CMV pp65 protein-derived control peptide, and used as target cells for Mart-1/HLA-A2 specific CTL in standard CTL assay. As shown in Fig 5C, Mart-1/HLA-A2 specific CTL efficiently lysed H441 in the presence of cognate peptides but not control peptide, and there was a significant increase in T cell mediated cytotoxicity when H441 was pretreated with EGFR inhibitor even at lower effector:target (5:1) ratio. Considering H441 is not the most sensitive cell line to EGFR inhibitor compared to ones with sensitive EGFR mutations (e.g. HCC827 or HCC4006), it is possible to extrapolate our results that EGFR inhibition could facilitate neo-antigen specific T cell responses and subsequent tumor killing in lung cancer especially with sensitive EGFR mutations.

### PD-L1 over-expressing lung cancer cell lines display inflammatory phenotype

**Fig 6:**
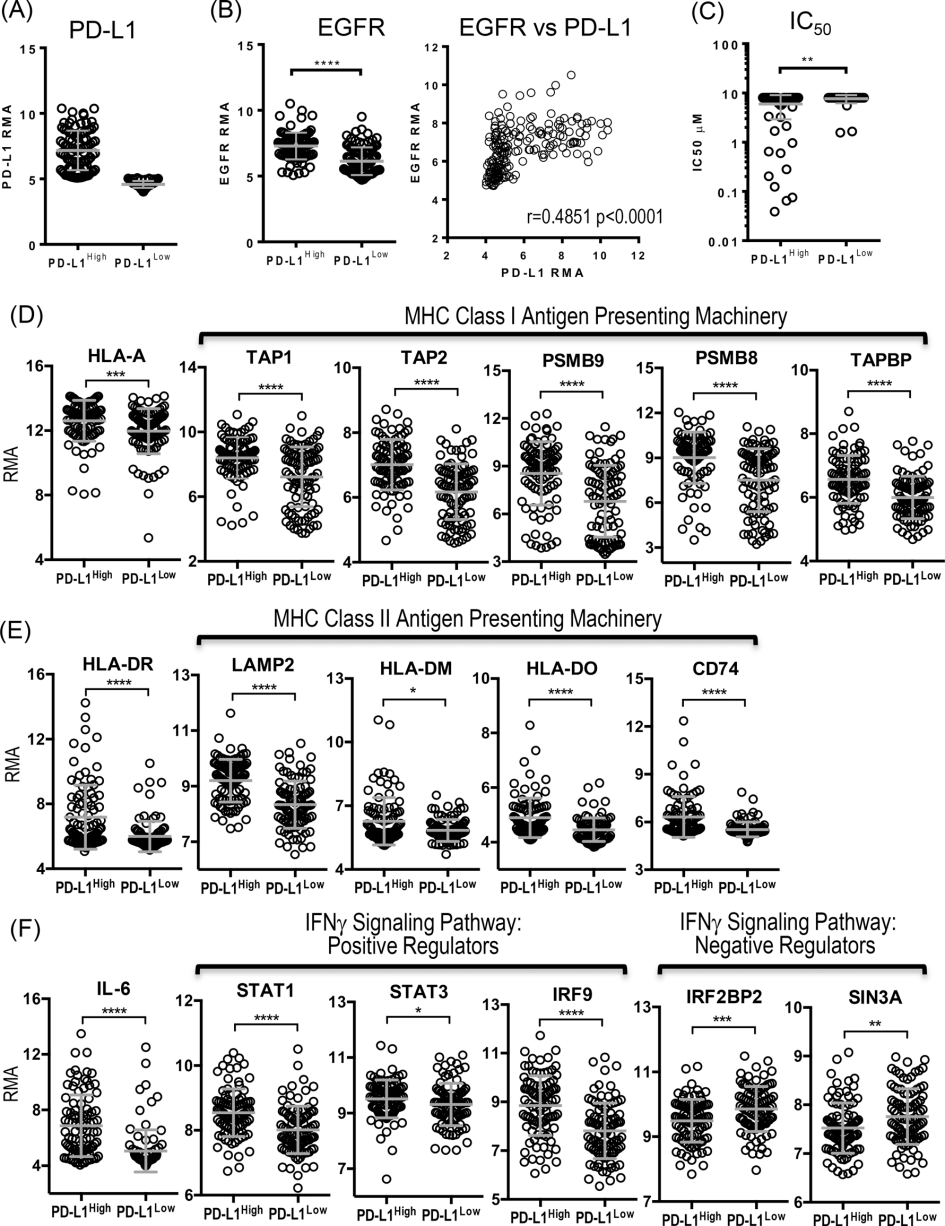
Overexpression of PD-L1 is associated with higher expression of EGFR and immune-related genes, and sensitivity to EGFR inhibitors. (A) Lung cancer cell lines (N186) from Cancer Cell Line Encyclopedia (CCLE) database were divided into two groups according to median expression of PD-L1 at Log_2_ Robust Multi-array Average (RMA), 5.087. Mean Log_2_ RMAs of PD-L1 expression of PD-L1^high^ and PD-L1^low^ groups were 7.171 ±1.508 and 4.578 ± 0.2630, respectively. (B) EGFR expression of PD-L1^high^ and PD-L1^low^ lung cancer cell lines, and correlation between EGFR and PD-L1 expression (C) The half maximum inhibitory concentration (IC_50_) of Erlotinib for PD-L1^high^ lung cancer cell lines (N51) and PD-L1^low^ lung cancer cell lines (N40). (D) Expression of MHC-I and genes associated with MHC-I antigen presentation from PD-L1^high^ and PD-L1^low^ lung cancer cell lines. (E) Expression of MHC-II and genes associated with MHC-II antigen presentation from PD-L1^high^ and PD-L1^low^ lung cancer cell lines. (F) Expression of genes associated with inflammation from PD-L1^high^ and PD-L1^low^ lung cancer cell lines. PD-L1^high^ lung cancer cell lines are associated with higher expression of EGFR and sensitivity to erlotinib (B and C). In addition, PD-L1^high^ lung cancer cell lines express genes associated with MHC-I and II antigen presenting pathways higher than PD-L1^low^ lung cancer cell lines (D and E). Significantly higher expression of IL-6 and positive regulators for IFNγ signaling pathway such as STAT1, STAT3, and IRF9 were associated with PD-L1^high^ lung cancer cell lines, while the expression of negative regulators for IFNγ signaling pathway such as IRF2BP2 and SIN3A were significantly lower in PD-L1^high^ lung cancer cell lines (F). P values were calculated by Wilcoxon-Man-Whitney test and summarized as follows, ****: *p* < 0.0001, ***: *p* = 0.0001 to 0.001, **: *p* = 0.001 to 0.01, *: *p* = 0.01 to 0.05, ns: *p* > 0.05.

As we observed a higher expression of antigen presentation molecules and PD-L1 especially in response to inflammatory signal, IFNγ, in lung cancer cell lines sensitive to EGFR inhibitors, we assessed the baseline expression of various immune-related genes in lung cancer cell lines from Cancer Cell Line Encyclopedia (http://www.broadinstitute.org/ccle) in relation to the PD-L1 expression. The gene expression profiles of 186 lung cancer cell lines were retrieved from CCLE database, and 186 lung cancer cell lines were divided into two groups, PD-L1^high^ and PD-L1^low^ according to the median expression value of PD-L1 (Fig 6A). PD-L1^high^ lung cancer cell lines were associated with a higher expression of EGFR compared to PD-L1^low^ lung cancer cell lines, suggesting that not only sensitive EGFR mutation but also overexpression of EGFR is associated with a higher baseline expression of PD-L1 (Fig 6B and Table 2). Additionally, the higher expression of EGFR in lung cancer cell lines correlated to the higher level of PD-L1 expression (Pearson’s correlation coefficient r = 0.4851 p<0.0001, Table 3). Drug sensitivity data to EGFR inhibitor that were available for 91 out of 186 lung cancer cell lines in CCLE database were compared between PD-L1^high^ and PD-L1^low^ lung cancer cell lines (Fig 6C). As expected, PD-L1^high^ lung cancer cell lines were more sensitive to the inhibition of EGFR signaling pathway compared to PD-L1^low^ lung cancer cell lines, and the inverse correlation was observed between overexpression of PD-L1 and IC50 value (Pearson’s correlation coefficient r = -0.2113, p = 0.0443,Table 3).

**Table 2:** The expression of immune-related genes associated with PD-L1^High^ vs PD-L1^Low^ expressing lung cancer cell lines from CCLE.

Gene	Function	PD-L1^high^ (N93) RMA* (mean ±SD)	PD-L1^low^ (N93) RMA* (mean ±SD)	P value
**CD274**	Program Death Receptor Ligand -1	7.171 ± 1.508	4.578 ± 0.2630	< 0.0001
**EGFR**	Epidermal Growth Factor Recepor	7.292 ± 1.015	6.135 ± 1.051	< 0.0001
**MHC I Antigen Presenting Pathway**				
**HLA-A**	HLA-A	12.60 ± 1.264	11.96 ± 1.394	0.0001
**CALR**	Calreticulin	8.329 ± 0.6181	8.121 ± 0.5782	0.0177
**TAP1**	Transporter associated with Antigen Processing 1	8.405 ± 1.277	7.224 ± 1.840	< 0.0001
**TAP2**	Transporter associated with Antigen Processing 2	7.011 ± 0.7768	6.174 ± 0.8587	< 0.0001
**PSMB9**	Proteasome Subunit Beta Type 9, LMP2	8.546 ± 2.000	6.799 ± 2.255	< 0.0001
**PSMB8**	Proteasome Subunit Beta Type 8, LMP7	9.014 ± 1.709	7.508 ± 2.101	< 0.0001
**TAPBP**	TAP binding protein	6.567 ± 0.7486	5.998 ± 0.6676	< 0.0001
**MHC II Antigen Presenting Pathway**				
**HLA-DRB**	HLA-DR	7.186 ± 1.983	5.995 ± 0.9363	< 0.0001
**LAMP2**	Lysosome-associated membrane protein 2	9.197 ± 0.7668	8.340 ± 0.8500	< 0.0001
**HLA-DM**	HLA-DM	6.270 ± 1.115	5.840 ± 0.5174	0.0398
**HLA-DO**	HLA-DO	4.895 ± 0.7233	4.458 ± 0.4282	< 0.0001
**CD74**	HLA-DR antigens-associated invariant chain	6.310 ± 1.284	5.526 ± 0.4654	< 0.0001
**Genes Associated with Inflammation**				
**IL6**	IL-6	6.868 ± 2.209	5.055 ± 1.521	< 0.0001
**IL6R**	IL-6 receptor	5.963 ± 0.9303	5.283 ± 0.8316	< 0.0001
**IL6ST**	IL-6 signal transducer	9.028 ± 1.072	7.550 ± 1.459	< 0.0001
**STAT1**	Signal transducer and activator of transcription 1	8.543 ± 0.7359	8.015 ± 0.7340	< 0.0001
**STAT3**	Signal transducer and activator of transcription 3	9.515 ± 0.6805	9.316 ± 0.7612	0.0228
**IRF1**	Interferon regulatory factor 1	7.086 ± 0.8899	6.172± 0.9304	< 0.0001
**IRF9**	Interferon regulatory factor 9	8.847 ± 1.223	7.815 ± 1.143	< 0.0001
**IRF2BP2**	Interferon regulatory factor-2 (IRF2) binding protein	9.492 ± 0.6546	9.854 ± 0.6941	0.0005
**SIN3A**	SIN3 transcription regulator family member A	7.526 ± 0.5169	7.758 ± 0.5702	0.0039
**Chemokines and Chemokine Receptors**				
**IL-8**	Neutrophil chemotactic factor	7.649 ± 2.516	5.946 ± 2.512	< 0.0001
**CXCL5**	Neutrophil chemotactic factor	5.523 ± 3.025	1.927 ± 0.1998	< 0.0001
**CXCL12**	Stromal cell-derived factor 1	5.523 ± 3.025	4.184 ± 1.927	0.5955
**CXCR1**	IL-8 receptor	5.414 ± 0.3376	5.281 ± 0.2860	0.0039
**CXCR4**	CXCL12 receptor	4.799 ± 1.436	6.438 ± 2.449	< 0.0001
**CXCR7**	CCL12 receptor	5.386 ± 2.138	6.195 ± 2.761	0.1505

*Gene expression signal was normalized using Robust Multi-array Average (RMA), and represented in log_2_ RMA.

**Table 3:** The correlation between expression of immune-related genes and PD-L1 of lung cancer cell lines from CCLE.

Gene	Function	Pearson’s Coefficient (r)	P value
**IC50**	The half maximum inhibitory concentration	-0.2113	0.0443
**EGFR**	Epidermal Growth Factor Recepor	0.4851	< 0.0001
**MHC I Antigen Presenting Pathway**			
**HLA-A**	HLA-A	0.2969	< 0.0001
**CALR**	Calreticulin	0.2032	0.0054
**TAP1**	Transporter associated with Antigen Processing 1	0.3723	< 0.0001
**TAP2**	Transporter associated with Antigen Processing 2	0.4894	< 0.0001
**PSMB9**	Proteasome Subunit Beta Type 9, LMP2	0.3971	< 0.0001
**PSMB8**	Proteasome Subunit Beta Type 8, LMP7	0.3943	< 0.0001
**TAPBP**	TAP binding protein	0.4860	< 0.0001
**MHC II Antigen Presenting Pathway**			
**HLA-DRB**	HLA-DR	0.2590	0.0004
**LAMP2**	Lysosome-associated membrane protein 2	0.3701	<0.0001
**HLA-DM**	HLA-DM	0.1055	0.1517
**HLA-DO**	HLA-DO	0.3666	< 0.0001
**CD74**	HLA-DR antigens-associated invariant chain	0.3225	< 0.0001
**Genes Associated with Inflammation**			
**IL6**	IL-6	0.4057	< 0.0001
**IL6R**	IL-6 receptor	0.3740	< 0.0001
**IL6ST**	IL-6 signal transducer	0.4853	< 0.0001
**STAT1**	Signal transducer and activator of transcription 1	0.3373	< 0.0001
**STAT3**	Signal transducer and activator of transcription 3	0.08563	0.2452
**IRF1**	Interferon regulatory factor 1	0.4212	< 0.0001
**IRF9**	Interferon regulatory factor 9	0.4716	< 0.0001
**IRF2BP2**	Interferon regulatory factor-2 (IRF2) binding protein	-0.2020	0.0057
**SIN3A**	SIN3 transcription regulator family member A	-0.2837	< 0.0001
**Chemokines and Chemokine Receptors**			
**IL-8**	Neutrophil chemotactic factor	0.3496	< 0.0001
**CXCL5**	Neutrophil chemotactic factor	0.2093	0.0042
**CXCL12**	Stromal cell-derived factor 1	0.0616	0.4031
**CXCR1**	IL-8 receptor	0.1663	0.0233
**CXCR4**	CXCL12 receptor	-0.4254	< 0.0001
**CXCR7**	CCL12 receptor	-0.1519	0.0385

As we showed that lung cancer cell lines sensitive to EGFR inhibitors expressed a trend towards higher baseline levels of MHC-I and PD-L1, and also higher expression of PD-L1, MHC-I, and MHC-II in response to IFNγ, we investigated whether PD-L1 overexpression is associated with overexpression of other immune-related genes in 186 lung cancer cell lines from CCLE database. The expression profiles of selected immune-related genes involved in antigen presentation, inflammation, and chemokines and their receptors were retrieved, and compared between PD-L1^high^ and PD-L1^low^ lung cancer cell lines (Fig 6D–6F and Table 2). PD-L1^high^ lung cancer cell lines showed a greater expression of selected genes involved in class I antigen presenting pathway (HLA-A, Calr, TAP1&2, PSMB9, PSMB8, TAPBP) and class II antigen presenting pathway (HLA-DR, LAMP2, HLA-DM, HLA-DO, and CD74) (Fig 6D and 6E), and inflammation such as IL-6 and STAT3, and several positive regulators of transcription factors involved in IFNγ signaling pathway (STAT1, IRF9) (Fig 6F) compared to PD-L1^low^ lung cancer cell lines. Again, the overexpression of PD-L1 proteins in lung cancer cell lines was correlated to the higher expression of genes associated with antigen presentation and inflammation, confirming our findings (Table 3). Interestingly, PD-L1^high^ lung cancer cell lines were associated with higher expression of certain chemokines such as IL-8 and CXCL5 (neutrophil chemotactic factors), and chemokine receptor such as CXCR1 (IL-8 receptor) while PD-L1^low^ lung cancer cell lines were associated with higher expression of CXCR4 and CXCR7 that are essential for lung cancer progression and angiogenesis (Tables 2 and 3) [38,39]. Our findings imply that EGFR driven lung cancer cell lines may have distinct inflammatory phenotype with a potential to elicit anti-tumor T cell responses.

## Discussion

Human keratinocytes are an essential component in the skin immune system as they can regulate both innate and adaptive immune responses through the production of various cytokines, chemokines, and anti-microbial peptides in response to skin injury, inflammation, and infections [40,41]. In addition, they can serve as non-professional antigen presenting cells through up-regulation of antigen presenting molecules–MHC-I and MHC-II in certain inflammatory conditions, e.g., in psoriasis, likely in response to IFNγ or other inflammatory cytokines such as TNFα [41–43]. Pollack et al investigated potential effects of EGFR inhibition on the expression of MHC-I, MHC-II, and CIITA complex of skin keratinocytes, and found that indeed EGFR inhibition augmented IFNγ induced expression of MHC-I, MHC-II, and CIITA complex, thus suggesting a potential interplay of autoreactive T cells in the mechanism of EGFR induced skin rash [18]. PD-L1, one of the prominent immune-check point proteins that play a crucial role in maintaining immune-tolerance under normal physiological condition is up-regulated by various inflammatory signals such as TNFα or IFNγ to prevent further tissue damage from excessive inflammation [44,45]. Such up-regulation of PD-L1 on keratinocytes in the area of inflammation is thought to protect the epidermis from damages by autoreactive T cells [46–48]. Although EGFR inhibition can synergistically up-regulate MHC-I and MHC-II on keratinocytes with IFNγ, whether or not it alters IFNγ induced PD-L1 expression on keratinocytes has not been previously reported. In the current study, we investigated the effects of the EGFR inhibitor, erlotinib, on the concurrent expression of MHC-I and MHC–II, and PD-L1 to define the potential impact of EGFR inhibition on autoreactive T cell responses to human skin keratinocytes. We found that EGFR inhibition did abrogate PD-L1 expression in IFNγ-treated human keratinocyte HaCaT cells, while augmenting the expression of MHC-I and MHC-II. These observations suggest that EGFR inhibition may promote the immune-mediated damage to skin keratinocytes by infiltrating autoreactive T cells in the affected skin, contributing to the immune-mechanism of skin rash caused by EGFR inhibition.

As the clinical benefit derived from the treatment with EGFR inhibitors is associated with the severity of the skin toxicity in lung and colorectal cancer, we hypothesized that in addition to the well-known cytotoxic effects of EGFR inhibition, a similar modulation of antigen presenting molecules and PD-L1 proteins by EGFR inhibition in tumor cells may be implicated in augmenting anti-tumor immune responses, thus contributing to an additional mechanism of anti-tumor activity in these cancers. Therefore, we investigated the impact of EGFR inhibition on the expression of antigen presenting molecules and PD-L1 proteins in lung cancer cell lines with a wide range of *in vitro* sensitivity to erlotinib, one of the three EGFR inhibitors that have been approved for the first line therapy of EGFR-mutated lung cancer.

First, we observed a distinct difference in the expression pattern of antigen presenting molecules and PD-L1 between lung cancer cell lines sensitive to erlotinib (EC_50_ <8) and those resistant to erlotinib (EC_50_ ≥8). There was a trend toward higher baseline surface expression of PD-L1 and MHC-I proteins in lung cancer cell lines sensitive to Erlotinib, and significantly increased expression of MHC-I, MHC-II, and PD-L1 in response to IFNγ in lung cancer cell lines sensitive to erlotinib when compared to those resistant to erlotinib. This is an interesting and important finding, suggesting that there might be a crosstalk between the EGFR and IFNγ receptor mediated signaling pathways that are essential in the transcriptional control of immune-related genes especially for classical antigen presenting pathway involving MHC-I and MHC-II.

Overexpression of PD-L1 has been implicated as a mechanism of immune-evasion in lung cancer as well as other solid tumors, and has been reported to be associated with the presence of sensitizing EGFR mutations in selected lung cancer cell lines [20–23] and resected non-small cell lung cancer [23–25]. Here, we confirmed a trend toward higher expression of PD-L1 proteins in lung cancer cell lines sensitive to EGFR inhibitors, and demonstrated for the first time that the expression of PD-L1 can be further up-regulated in the presence of IFNγ especially in lung cancer cell lines sensitive to EGFR inhibitors. Further, the analysis of gene expression dataset of 186 lung cancer cell lines from CCLE revealed that the overexpression of PD-L1 is associated with higher expression of EGFR and sensitivity to EGFR inhibitors, higher expression of genes related to MHC-I and MHC-II antigen presenting pathways, inflammation and IFNγ signaling pathway, and chemokines for certain inflammatory immune cells such as neutrophils. Our findings indicate that PD-L1^high^ lung cancer cell lines display a pro-inflammatory phenotype with great potential for neo-antigen presentation and recruiting immune cells that are essential to initiate anti-tumor immunity. As the overexpression of PD-L1 in EGFR-driven lung cancer cells may be a mechanism of immune-tolerance to evade the tumor killing by infiltrating immune cells, the PD-1 and PD-L1 axis can be a reasonable therapeutic target to break the immune-tolerance and to enhance anti-tumor immunity in EGFR-driven lung cancer.

Blockade of PD-1/PD-L1 axis is the novel immunotherapeutic modality with a striking clinical efficacy in the treatment of many solid tumors such as melanoma, renal cell carcinoma, non-small cell lung cancer, and certain malignancies with mismatch-repair deficiency [26–29]. Anti-PD1/PD-L1 blocking strategies appear to be more effective for the treatment of lung cancers in patients with a history of smoking[49], which comprise a very small proportion of EGFR-mutated lung cancers but a significant proportion of tumors with high while type EGFR expression and high mutation density when compared to tumors that arise in oligo-smokers [50,51]. As we demonstrated that there is a positive correlation between EGFR expression and PD-L1 expression status, the overexpression of EGFR may be a determinant of treatment response to immunotherapy targeting PD1/PD-L1 axis in the lung cancers that arise in these smoking population. Thus, the EGFR overexpression as a predictive biomarker of response to in PD1/PD-L1 blocking strategies warrants further investigations.

The presence of intratumoral infiltrating PD-1^high^ CD8^+^ T cells and their subsequent expansion upon PD-1/PD-L1 blockade predicts the effective tumor regression, suggesting the direct role of anti-tumor T cell responses in immunotherapy strategies that target the PD-1/PD-L1 axis [21,52]. EGFR inhibitors have been previously reported to down-regulate PD-L1 expression in lung cancer cell lines carrying sensitizing EGFR mutations, suggesting that they may potentially function as immune-modulators in addition to their direct cytotoxic effects on tumor cells [21,22]. Here, we extend current knowledge by demonstrating that down-regulation of PD-L1 by EGFR inhibitors is not restricted to lung cancer cell lines carrying sensitizing EGFR mutations, but also observed in lung cancer cell lines with wild type EGFR in a wide range of sensitivity to EGFR inhibitors (e.g. H441), and that EGFR inhibitors abrogate the IFNγ-induced overexpression of PD-L1 without adversely affecting the expression of antigen presenting molecules in lung cancer cell lines. Further, we were able to show that EGFR inhibition can indeed enhance antigen specific T cell mediated tumor killing. Thus, our findings suggest that the EGFR inhibitors may potentially facilitate anti-tumor adaptive immune responses by breaking tolerance especially in lung cancers with sensitizing EGFR mutations or EGFR overexpression that are associated with over-expression of PD-L1.

In summary, we seek to elucidate a novel immune-mechanism of EGFR inhibitor-induced skin rash and corresponding anti-tumor immunity in lung cancer. First, we demonstrated that PD-L1 overexpression in lung cancer is associated with increased sensitivity to EGFR inhibitors, and a higher expression of EGFR and genes critical to neo-antigen presentation, and that down-modulation of PD-L1 may be an additional mechanism of anti-tumor activity of EGFR inhibitors in augmenting anti-tumor T cell responses. Thus, our study provides a rationale for conducting clinical studies to investigate combination therapy with EGFR inhibitors and PD1/PD-L1 axis blocking antibodies especially in EGFR-driven lung cancer with either as a result of sensitizing mutations or overexpression of wild type EGFR.
